# Delayed apoptosis allows islet β-cells to implement an autophagic mechanism to promote cell survival

**DOI:** 10.1371/journal.pone.0172567

**Published:** 2017-02-17

**Authors:** Heather L. Hayes, Brett S. Peterson, Jonathan M. Haldeman, Christopher B. Newgard, Hans E. Hohmeier, Samuel B. Stephens

**Affiliations:** 1 Sarah W. Stedman Nutrition and Metabolism Center, Duke University Medical Center, Durham, North Carolina, United States of America; 2 Duke Molecular Physiology Institute, Duke University Medical Center, Durham, North Carolina, United States of America; 3 Department of Pharmacology and Cancer Biology, Duke University Medical Center, Durham, North Carolina, United States of America; 4 Department of Medicine, Division of Endocrinology, Duke University Medical Center, Durham, North Carolina, United States of America; University of Bremen, GERMANY

## Abstract

Increased β-cell death coupled with the inability to replicate existing β-cells drives the decline in β-cell mass observed in the progression of both major forms of diabetes. Understanding endogenous mechanisms of islet cell survival could have considerable value for the development of novel strategies to limit β-cell loss and thereby promote β-cell recovery. Insulinoma cells have provided useful insight into β-cell death pathways but observations made in cell lines sometimes fail to translate to primary islets. Here, we report dramatic differences in the temporal regulation and engagement of the apoptotic program in primary rodent islets relative to the INS-1 derived 832/13 cell line. As expected, 832/13 cells rapidly induced cell stress markers in response to ER stress or DNA damage and were fully committed to apoptosis, resulting in >80% cell death within 24 h. In contrast, primary rat islets were largely refractory to cell death in response to ER stress and DNA damage, despite rapid induction of stress markers, such as XBP-1(s), CHOP, and PUMA. Gene expression profiling revealed a general suppression of pro-apoptotic machinery, such as Apaf-1 and caspase 3, and sustained levels of pro-survival factors, such as cIAP-1, cIAP-2, and XIAP, in rat islets. Furthermore, we observed sustained induction of autophagy following chronic ER stress and found that inhibition of autophagy rendered islet β-cells highly vulnerable to ER stress-induced cell death. We propose that islet β-cells dampen the apoptotic response to delay the onset of cell death, providing a temporal window in which autophagy can be activated to limit cellular damage and promote survival.

## Introduction

Pancreatic islets coordinate changes in fuel availability and energy demand via release of the glucoregulatory hormones insulin and glucagon. Insulin insufficiency due to loss of islet β-cell mass and function coupled with increasing peripheral (fat, muscle, and liver) insulin resistance leads to sustained hyperglycemia and ultimately the development of Type 2 diabetes [1]. Loss of β-cell mass stems from increased β-cell death [2, 3] and possibly de-differentiation of β-cells into endocrine progenitor cells [4]. Although significant efforts have been made to restore β-cell mass via stimulating β-cell replication, the poor regenerative capacity of the adult β-cell remains a significant obstacle for diabetes treatment [5–7]. Thus, understanding β-cell survival pathways may prove crucial to promoting the maintenance of functional islet β-cell mass and preventing further disease progression.

Programmed cell death, or apoptosis, is a physiological mechanism used to regulate cell numbers and eliminate undesirable cell populations. Cells undergo apoptosis for a variety of reasons that include tissue remodeling during development, turnover of actively dividing populations such as the gut epithelium, and the removal of damaged cells that may accumulate oncogenic mutations. In the adult animal, the ability to repopulate or replenish lost cell mass influences the extent to which damaged cells will undergo apoptosis [8]. For example, the removal of damaged gut epithelial cells via apoptosis is favored over survival because these cells can be easily replaced through active cell division. In contrast, cell populations with limited regenerative capacity, such as cardiomyocytes and neurons, promote survival over death because these cells are not easily replaced. To promote survival, neurons and cardiomyocytes utilize a number of mechanisms to circumvent the apoptotic cascade. For example, increased levels of the CARD domain-containing inhibitors of apoptosis (IAPs), such as XIAP, and reduced levels of Apaf-1 are utilized to suppress caspase activation and prevent apoptosis in neurons [9–12]. This allows cells sufficient time to mitigate the impact of cellular damage [13, 14]. Whether such mechanisms occur in primary β-cells, which are also limited in their regenerative ability, is not known.

Autophagy is a cellular recycling program that utilizes lysosomal degradation to promote turnover of long-lived proteins and cytoplasmic organelles [15]. Increasing evidence demonstrates a critical role for autophagy in regulating β-cell health and function. Early studies identified a form of microautophagy, known as crinophagy, as a key mechanism for turnover of insulin granules [16]. Consistent with this, β-cell specific loss of autophagic components, such as Atg7, result in hypoinsulinemia and subsequent hyperglycemia [17]. Further activation of autophagy may also be an important coping mechanism in β-cell stress. An increase in autophagosome density has been described in β-cells in multiple rodent models of diabetes [17–21] and in human T2D subjects [22]. Furthermore, β-cell knockout of Atg7 increases β-cell loss and accelerates diabetes onset in rodent models [18–21, 23]. Thus, understanding the role of autophagy in β-cell survival may offer a unique entry point for identifying novel diabetes targets.

In this report, we investigated β-cell apoptotic pathways using the rat insulinoma-derived cell line, 832/13, and primary rat islets. We demonstrate significant differences in the extent and temporal regulation of cell death in primary islets and insulinoma cells induced by ER stress and DNA damage. While insulinoma cells typically undergo apoptosis within 24 h of cell stress, primary islets require up to 72 h for a significant rise in cell death despite early activation of conserved cell stress pathways. Moreover, we demonstrate that primary islets possess the ability to recover β-cell function following brief (24 h) ER stress exposure. The delay in islet cell death was associated with a general reduction in the expression of the apoptosis machinery (Apaf-1 and caspase 3) and either sustained or elevated expression of IAPs; however, overexpression of terminal apoptotic factors (Apaf-1, caspase 3 and caspase 9) was not sufficient to increase islet cell apoptosis rates. Rather, we show that activation of autophagy following ER stress is required for β-cell survival and that suppression of the autophagic response renders islet β-cells unable to survive an ER stress insult, resulting in rampant cell death. These data suggest that primary β-cells dampen their apoptotic response which allows an autophagic program sufficient time to limit cell damage and enhance β-cell survival in response to cytotoxic agents.

## Materials and methods

### Cell culture and reagents

INS-1-derived 832/13 rat insulinoma cells were cultured as previously described [24]. Pancreatic islets were isolated from male Wistar rats at 12–16 weeks of age and cultured as previously described [25–27] under a protocol approved by the Duke University Institutional Animal Care and Use Committee. Cell culture reagents were from Thermo Life Technologies unless specified otherwise. Chemical reagents were from Sigma-Aldrich unless specified otherwise. Plasmid containing cDNA encoding human Apaf-1 (Transomics Clone; BC136532) was PCR amplified and subcloned into pDONR221 (Thermo Life) with BP Clonase (Thermo Life) per the manufacturer’s protocol. Plasmids containing cDNAs encoding caspase 3 (HsCD00043626) and caspase 9 (HsCD00043776) were from DNASU [28]. cDNAs were recombined into pAd/CMV/V5-DEST (Thermo Life) using LR Clonase II plus (Thermo Life) per the manufacturer’s protocol [29]. Recombinant adenoviruses were generated in HEK293 cells and purified by cesium chloride gradients. All recombinant viruses were determined to be E1A deficient using a quantitative PCR screen [30]. Pools of islets were transduced with ~2 x 10^7^ IFU/ mL adenovirus (MOI ~100–200) for 18 h and assayed 72 h post-treatment. 832/13 cells were transduced with ~2 x 10^6^ IFU/mL adenovirus (MOI ~10–20) for 18 h and assayed 72 h post-treatment.

### Immunoblot analysis

Insulinoma cells and primary islets were harvested and lysed in ice-cold RIPA buffer containing protease inhibitors (BD biosciences). Clarified cell lysates were resolved on 4–12% NuPAGE gels (Invitrogen) and transferred to polyvinylidene difluoride (PVDF) membranes. Membranes were probed with antibodies raised against the following proteins: Apaf-1 (R&D Systems Cat# MAB868), caspase 3 (Cell Signaling Technology Cat# 9662), human caspase 9 (Cell Signaling Technology Cat# 9502, RRID:AB_2068621), CHOP (Cell Signaling Technology Cat#2895), LC3B (Cell Signaling Technology Cat#2775), PARP (Cell Signaling Technology Cat# 9542, RRID:AB_2160739), XIAP (R&D Systems Cat#AF8221), and γ-tubulin (Sigma-Aldrich Cat# T6557, RRID:AB_477584). Primary antibodies were detected using goat anti-mouse IRDye 800CW (Li-COR) or Alexa Fluor 680 goat anti-rabbit IgG (Thermo Life Technologies) secondary antibodies. Immunoblots were developed using an Odyssey CLx system (Li-COR).

### Caspase 3/7 activity

832/13 cells and primary rat islets were lysed in ice-cold lysis buffer (50 mM HEPES pH 7.2, 0.1% CHAPS, 0.1 mM EDTA, and 1 mM DTT) containing protease inhibitors (BD). Clarified lysates were mixed with assay buffer (50 mM HEPES pH 7.2, 0.1% CHAPS, 1 mM EDTA, 100 mM NaCl, 10% glycerol, and 10 mM DTT) at a 1:1 ratio. The caspase 3/7 substrate, Ac-DEVD-pNA substrate (ENZO Life Sciences), was then added to the mixture, and caspase 3/7 activity was detected at 405 nm using a Spectromax M5 microplate reader at 37°C for up to 1 h.

### Annexin V staining

832/13 cells were lifted and stained live on ice with Annexin V (Invitrogen) or propidium iodide according to the manufacturer’s instructions. Pools of 50 islets were dispersed with trypsin/EDTA (0.025%) and stained with Annexin V (Invitrogen) or propidium iodide. Data were collected using a FACscan analyzer and analyzed using WinMDI 2.9 software.

### Immunofluorescence

832/13 cells were treated with thapsigargin (100 nM), etoposide (100 μM), or vehicle (DMSO) for 24 h and plated on poly-D-lysine-coated coverslips (BD). Pools of 50 islets were treated with thapsigargin (1 μM), etoposide (200 μM), or vehicle (DMSO) for the indicated times. Islets were dispersed with trypsin/EDTA and attached to poly-D-lysine coated coverslips (BD) as previously described [31]. Primary antibodies raised against insulin (guinea pig; Dako) and CHOP (mouse; Cell Signaling) were incubated overnight and detected using species specific secondary antibodies coupled to AlexaFluor 488 (anti-guinea pig; Life Technologies) or AlexaFluor 647 (anti-mouse; Life Technologies). For detection of apoptosis, cells were stained using the *In situ* Cell Death detection kit (Roche). Cells were counterstained with DAPI. Images were captured on a Zeiss Axioplan 2 microscope using OpenLab software. Five to seven images containing approximately 200 nuclei per image per slide were evaluated for TUNEL staining using Fiji software. The percentage of TUNEL positive cells was determined as the ratio of TUNEL positive cells to total nuclei x 100.

In a separate series of studies, islets were dispersed with trypsin/EDTA and plated in 96-well plates pre-coated with extracellular matrix proteins from HTB9 cells as previously described [32] at a density of 7500 cells per well. After cell attachment, islet cells were treated with vehicle, chloroquine (5 μM), bafilomycin A1 (100 nM) alone or in combination with thapsigargin (1 μM) for 24–72 h. Cells were stained as described above (insulin and TUNEL) and imaged using a high content ThermoFisher ArrayScan at ZenBio, Inc. and analyzed using Cellomics software to identify distinct cell populations.

### Measurement of RNA levels

Total RNA was isolated using the RNeasy kit (Qiagen), and cDNA was synthesized using iScript (Bio-Rad). Real-time PCR assays were performed using the Viia7 detection system and software (Applied Biosystems). Primer sequences are available upon request. All mRNA levels were normalized to the expression in 832/13 cells via the comparative (Livak) C_t_ method (2^-ΔΔCt^) using PPIB as the control housekeeping gene, which remained consistent between tissues based on comparisons to other housekeeping mRNAs such as Rps9 (data not shown).

### Glucose-stimulated insulin secretion

Insulin secretion was measured in pools of 20 islets by static incubation in secretion assay buffer (SAB) containing 2.5 mM glucose for 1 h at 37°C (basal) followed by incubation in SAB containing 16.7 mM glucose for 1 h (stimulatory) as previously described [31]. Insulin was measured in SAB via ELISA (ALPCO). Cells were lysed in RIPA buffer and total protein determined by BCA (ThermoFisher). Insulin content was determined from whole cell lysates by ELISA.

### Statistical analysis

Data are presented as means + standard errors of mean (SEM) and graphed using GraphPad Prism software. Data were analyzed by ANOVA with Tukey post-hoc analysis for multiple group comparisons. A p value of < 0.05 was considered statistically significant.

## Results

### Primary rat islets are apoptosis resistant whereas insulinoma cells are apoptosis sensitive

**Fig 1:**
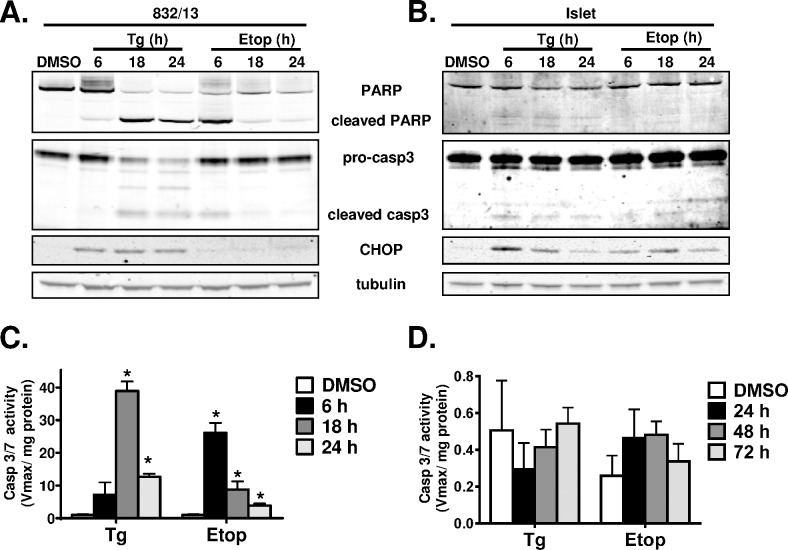
Caspase 3 activity is low in primary rat islets in response to ER stress and DNA damage. 832/13 cells (**A**, **C**) and primary rat islets (**B**, **D**) were treated with DMSO (control), thapsigargin (100 nM or 1 μM, respectively), or etoposide (100 μM or 200 μM, respectively) for the indicated times. Clarified lysates were examined by immunoblot analysis (**A**, **B**) and caspase 3/7 colorimetric activity assay (**C**, **D**). Data represent the mean +S.E.M of 3 independent experiments. * p ≤ 0.05 as compared to DMSO treated cells.

Much of the available data for understanding β-cell apoptosis has been generated using insulinoma cell lines. However, recent evidence suggests key differences exist in the temporal regulation of primary islet cell death compared to insulinoma cell culture models in response to cytotoxic stresses [33]. Here, we investigated the induction of apoptosis in the INS-1 derived subclone 832/13 as compared to primary rat islets in response to ER stress and DNA damage using the ER Ca^2+^-ATPase inhibitor, thapsigargin, and the topoisomerase II inhibitor, etoposide, respectively. As shown in Fig 1A, treatment of 832/13 cells with either thapsigargin or etoposide resulted in rapid generation (within 6 h) of the active (cleaved) form of caspase 3 and subsequent cleavage of the caspase 3 substrate poly (ADP-ribose) polymerase (PARP). Consistent with this data, we observed a robust increase in caspase 3/7 activity in 832/13 cells within 18 h of thapsigargin treatment and as early as 6 h after etoposide treatment (Fig 1C). In contrast, primary rat islets treated with thapsigargin or etoposide showed only faint levels of active (cleaved) caspase 3 by immunoblot analysis, and PARP cleavage was barely detectable in primary islets despite clear induction of the pro-apoptotic stress transcription factor C/EBP homologous protein (CHOP) (Fig 1B). Moreover, caspase 3/7 activity was indistinguishable from untreated control islets even after a 72 h treatment with apoptotic agents (Fig 1D).

**Fig 2:**
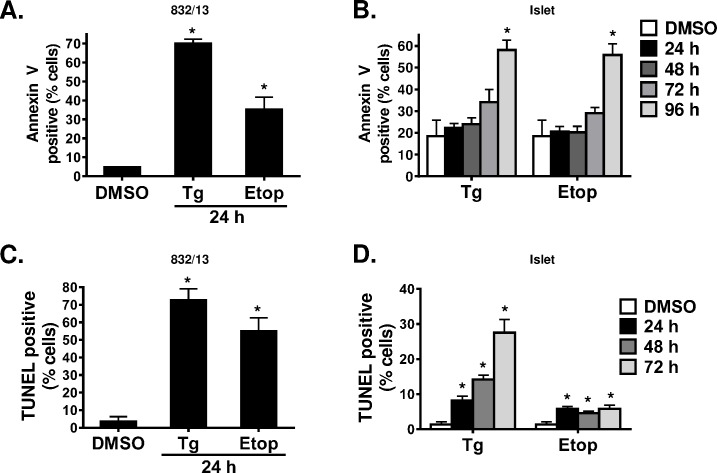
Early and late apoptotic marker analysis demonstrates low levels of apoptosis in primary rat islets. 832/13 cells (**A**, **C**) and primary rat islets (**B**, **D**) were treated with DMSO (control), thapsigargin (100 nM or 1 μM, respectively), or etoposide (100 μM or 200 μM, respectively) for the indicated times. (**A**, **B**) Cells were stained with Annexin V and counted by flow cytometry. (**C**, **D**) Cells were dispersed onto coverslips and stained with TUNEL and counterstained with DAPI. Positive cells were counted using Fiji. Data represent the mean +S.E.M. of 3 independent experiments. * p ≤ 0.05 as compared to DMSO treated cells.

To further evaluate cell death in 832/13 cells and rat islets, we used Annexin V staining as an early indication of apoptosis and TUNEL staining as a late apoptosis marker. In 832/13 cells, etoposide and thapsigargin elicited a robust increase in the number of Annexin V-positive (Fig 2A) and TUNEL-positive (Fig 2C) cells within 24 h of treatment. In contrast, significant accumulation of Annexin V-positive primary islet cells was only evident following 96 h treatment with thapsigargin or etoposide (Fig 2B). Similarly, a few islet cells (< 10%) were TUNEL-positive following 24 h treatment with apoptosis inducers (Fig 2D). This percentage increased to 30% after 72 h thapsigargin treatment but remained at 10% in the etoposide-treated islets. Consistent with our TUNEL staining data, propidium iodide (PI) staining also failed to detect considerable cell death in thapsigargin-treated islets (S1 Fig). Together, these data demonstrate a significant delay in the progression to apoptosis in primary rat islets as compared to insulinoma cells.

### Activation of a cell stress program is maintained in primary rat islets

**Fig 3:**
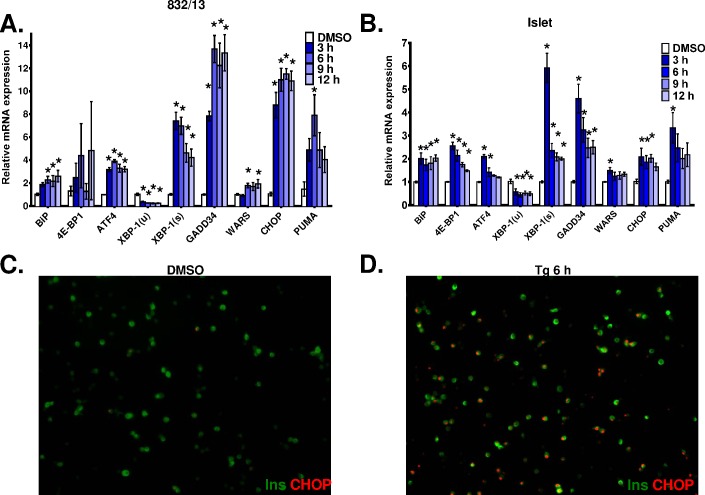
Similar induction of stress markers following thapsigargin treatment in 832/13 cells and primary rat islets. 832/13 cells (**A**) and primary rat islets (**B**) were treated with DMSO (control) or thapsigargin (100 nM or 1 μM, respectively) for the indicated times. mRNA expression levels were determined via qRT-PCR. Data represent the mean +S.E.M. (n = 3–4). * p ≤ 0.05 as compared to DMSO treated cells. Primary rat islets were treated with DMSO control (**C**) or thapsigargin (1 μM; **D**) for 6 h and dispersed onto coverslips. Cells were stained for insulin (green) and CHOP (red).

We hypothesized that the reduced level of islet cell apoptosis may be due to dampened or absent activation of cellular stress responses. Here, we examined the temporal induction of canonical cell stress markers elicited via ER stress. As shown in Fig 3A, treatment of 832/13 cells with thapsigargin elicited a rapid increase in ATF4, CHOP, and PUMA expression within 3 h that persisted throughout the 12 h time course. We also observed a strong increase in the level of spliced XBP-1(s) and a corresponding decrease in unspliced XBP-1(u). Downstream targets of CHOP, including the regulatory subunit of the eIF2α phosphatase GADD34 and the trytophanyl tRNA synthetase (WARS), were upregulated. In addition, an XBP-1(s) target gene, the ER resident chaperone BiP, was elevated. Similar to insulinoma cells, primary rat islets treated with thapsigargin readily increased expression of ER stress markers, namely XBP-1(s), CHOP, and PUMA (Fig 3B). Although the overall magnitude of induction of these markers was reduced compared to the insulinoma cell line, activation of key downstream target genes, such as GADD34 and BiP, was maintained. Consistent with the increase in CHOP target gene expression, the majority of islet cells treated with thapsigargin showed nuclear accumulation of CHOP within 6 h of treatment (Fig 3C and 3D). Furthermore, insulinoma cells and primary rat islets treated with etoposide exhibited similar induction of PUMA expression (data not shown). These data demonstrate that primary islets, like insulinoma cells, rapidly activate a stress response program when treated with inducers of ER stress and DNA damage.

**Fig 4:**
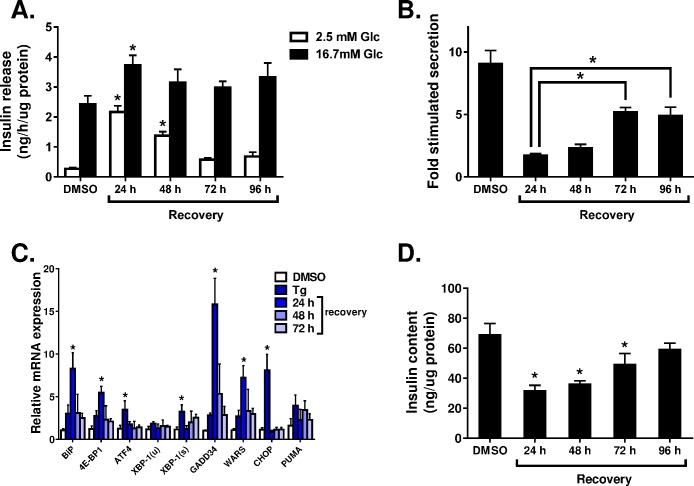
β-cells recover function following 24 h ER stress exposure. Primary rat islets were treated with thapsigargin (1 μM) for 24 h and allowed to recover for the indicated times. Glucose-stimulated insulin secretion (**A**) was measured by static incubation in media containing 2.5 mM glucose and 16.7 mM glucose for 1 h each and normalized to total protein. (**B**) Fold stimulation was determined as the ratio of insulin release at stimulatory (16.7 mM) versus basal (2.5 mM) glucose. (**C**) mRNA expression levels were determined via qRT-PCR. (**D**) Insulin content was determined from whole cell lysates. Data represent the mean +S.E.M of at least 3 independent experiments. * p < 0.05 as compared to the appropriate basal or stimulatory glucose in the DMSO (control) (**A**), or DMSO (control) treatment (**B**, **C**, **D**).

Given the ability of islets to resist apoptosis induction following prolonged stress (Figs 1 and 2), we examined the ability of islets to recover function from cellular damage. Here, rat islets were treated with thapsigargin overnight (24 h) and allowed to recover in growth media for up to 96 h. Islet β-cell function was assessed via glucose-stimulated insulin secretion (GSIS). Notably, this level of ER stress exposure was sufficient to induce apoptosis in the majority of insulinoma cells (Figs 1 and 2). As shown in Fig 4A, β-cell function was negatively impacted by ER stress with a large increase in insulin release at basal glucose (2.5 mM) for up to 48 h post-recovery. However, by 72 h, β-cell function improved with an increase in the fold-response to glucose (Fig 4B), which was driven by normalization of insulin release at basal glucose (Fig 4A) and restoration of insulin content (Fig 4D). Consistent with this, we observed mRNA expression levels of ER stress components returning to basal levels within 48 h of recovery from thapsigargin treatment (Fig 4C). These data suggest that primary islet β-cells have the capacity to recover from ER stress-induced cellular damage.

### Reduced expression of apoptotic machinery in primary islets

**Fig 5:**
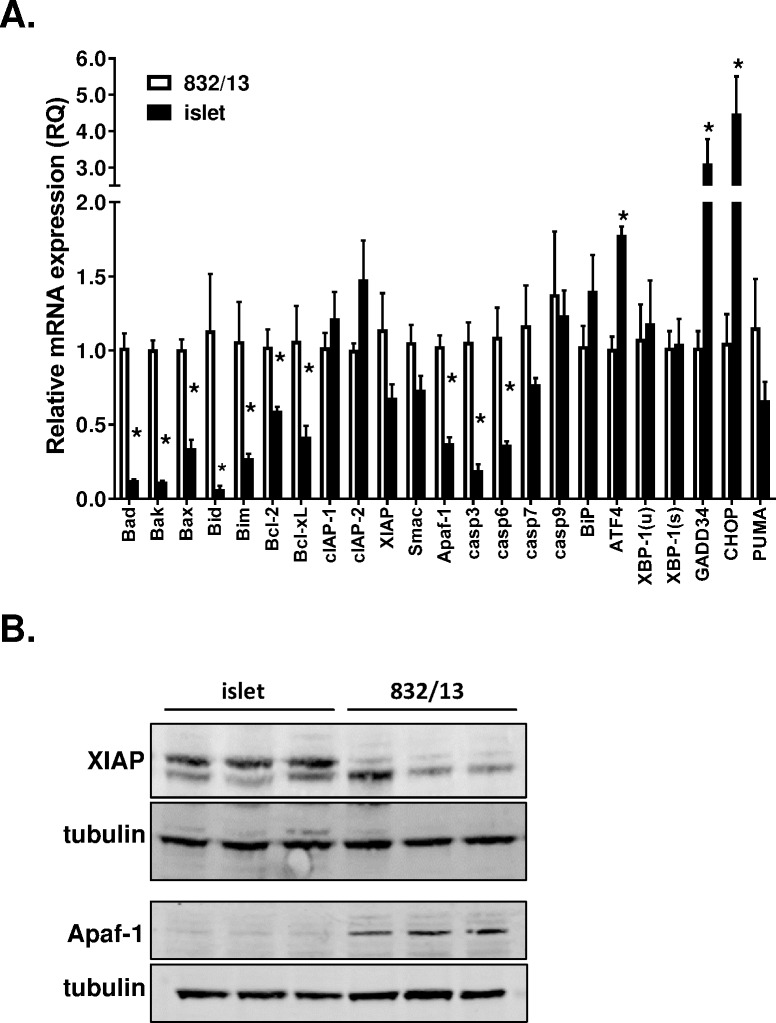
Expression of pro- and anti-apoptotic mRNAs is muted in primary rat islets. (**A**) Relative mRNA expression levels in primary islet cells were evaluated by qRT-PCR and compared to levels in 832/13 cells. Data represent the mean +S.E.M. (n = 3–5). * p < 0.05 as compared to 832/13 cells. (**B**) Immunoblot analysis of whole cell lysates.

Having established that primary rat islets demonstrate immediate responses to cell stress yet largely resist cell death, we next compared the relative expression levels of key apoptotic mediators in 832/13 cells and primary rat islets using quantitative RT-PCR. Rat islets expressed 70–80% less mRNA encoding the BH3-only containing pro-apoptotic factors Bad, Bak, Bax, Bid, and Bim compared to 832/13 cells (Fig 5A). Similarly, the anti-apoptotic Bcl-2 members, Bcl-2 and Bcl-xL, were reduced by 50–60% in primary rat islets compared to the insulinoma cell line. Core components of the apoptotic machinery, including the effector caspase 3 and the apoptosome scaffolding protein Apaf-1, were also reduced in rat islets compared to insulinoma cells. Additionally, caspases 8 and 12, though detected, were expressed at significantly lower levels than caspases 3, 6, 7, and 9 in both cell systems (data not shown). In contrast to the pro-apoptotic machinery, the CARD domain-containing inhibitors of apoptosis (IAPs), cIAP-1 and cIAP-2, and XIAP, were expressed at similar levels in 832/13 cells and primary islets.

Consistent with our mRNA expression data, we also observed considerable differences in expression of XIAP and Apaf-1 protein in 832/13 cells as compared to primary rat islets. As shown in Fig 5B, Apaf-1 was well-expressed in 832/13 cells yet detected only faintly in isolated islets. Conversely, XIAP protein levels were well-maintained in isolated islets and 832/13 cells. These data highlight key differences in the expression levels of pro- and anti-apoptotic components in insulinoma cells and primary rat islets.

In contrast to the pro-apoptotic factors assayed, components of the cell stress programs were not reduced in primary islets under normal (non-stress) growth conditions (Fig 5A). The levels of XBP-1(u), XBP-1(s), PUMA, and ATF4 mRNA were similar or slightly elevated in islets relative to insulinoma cells. Basal levels of CHOP and its downstream target GADD34 were consistently elevated by 3-4-fold in islets, suggesting that persistent activation of this pathway may occur in cultured islets. These data support the notion that islet cells have relatively low rates of apoptosis in response to cytotoxic stress as compared to insulinoma cells yet maintain a functional cell stress activation program.

**Fig 6:**
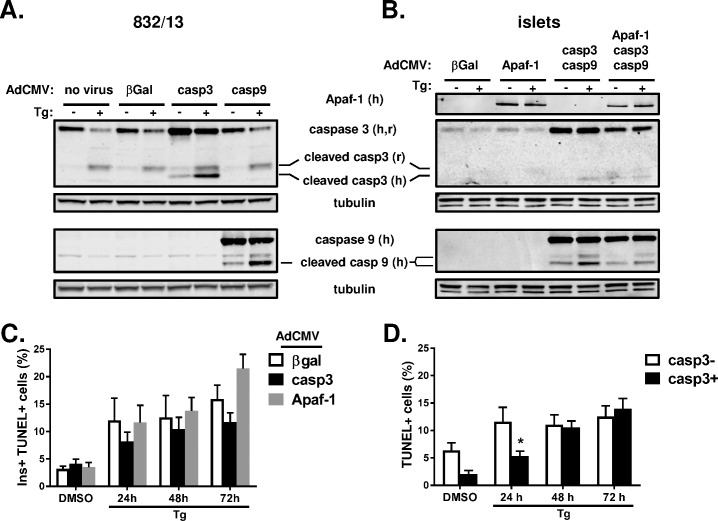
Overexpression of caspases is not sufficient to accelerate islet cell apoptosis. 832/13 and primary rat islets were untreated (no virus) or treated with recombinant adenoviruses containing cDNAs corresponding to human Apaf-1, human caspase 3, human caspase 9 or the control gene, β-galactosidase (β-gal), as indicated. 72 h post-infection, 832/13 cells (**A**) and primary rat islets (**B**) were treated with DMSO (control) or thapsigargin (100 nM or 1 μM, respectively) for 18 h. Clarified lysates were examined by immunoblot analysis. Endogenous rat (r) and overexpressed human (h) proteins are labeled accordingly. Of note, Apaf-1 and caspase 9 antibodies only detect human (overexpressed) proteins. (**C**, **D**) 72 h post adenoviral infection, rat islets were treated with DMSO (control) or thapsigargin (1 μM) for up to 72 h. Islet cells were stained for TUNEL, caspase 3, and insulin and counterstained with DAPI. Cells were imaged using a high content imager and analyzed using Cellomics software. (**C**) Percentages of insulin+ TUNEL+ islet cells are shown. (**D**) Comparison of the percentages of caspase 3+ (overexpressing), TUNEL+ islet cells vs. caspase 3- (non-overexpressing), TUNEL+ islet cells are shown. (**C**, **D**) Data represent the mean +S.E.M. of 3 independent experiments. * p ≤ 0.05 as compared to DMSO treated cells.

We next investigated if overexpression of terminal components of the apoptotic caspase cascade could accelerate islet cell apoptosis using recombinant adenoviruses (Fig 6). As expected, treatment of 832/13 cells with thapsigargin resulted in a clear increase in endogenous (rat) caspase 3 cleavage independent of adenoviral-mediated overexpression (Fig 6A). In addition, thapsigargin treatment of 832/13 cells overexpressing human caspase 3 demonstrated a clear increase in (human) caspase 3 cleavage as compared to control cells expressing β-galactosidase (β-gal). Similarly, overexpression of human caspase 9 in 832/13 cells resulted in a substantial increase in (human) caspase 9 cleavage following overnight thapsigargin treatment, although endogenous (rat) caspase 3 cleavage was not affected. In contrast, rat islets treated overnight with thapsigargin had considerably less detectable endogenous (rat) caspase 3 cleavage (Fig 6B) as compared to 832/13 cells (Fig 6A), consistent with our previous data (Fig 1). Rather strikingly, caspase 3 cleavage (rat and human) remained extremely low in primary islets overexpressing a combination of either caspases 3 and 9, or a combination of caspases 3, 9 and Apaf-1 as compared to islets treated with β-galactosidase (β-gal) control virus (Fig 6B). Interestingly, we did observe an increase in caspase 9 (human) cleavage with thapsigargin treatment, though this did not result in increased caspase 3 cleavage (rat or human). Consistent with the lack of caspase 3 cleavage in primary islets, we did not observe any differences in the accumulation of TUNEL-positive β-cells in islets overexpressing either Apaf-1 or caspase 3 treated with thapsigargin for up to 72 h (Fig 6C). Furthermore, because the adenoviral transduction efficiency is less than 100% in primary rat islets, we also considered the level of apoptosis in the specific population of β-cells overexpressing caspase 3. As shown in Fig 6D, we observed no increase in the extent or kinetics of islet cell apoptosis assessed by TUNEL staining in caspase 3-overexpressing primary islet cells (casp3+) as compared to non-caspase 3-overexpressing islet cells (casp3-) derived from the same total islet cell population. These data demonstrate that primary β-cells are resistant to induction of apoptosis even when key components of the apoptotic machinery are overexpressed.

### β-cells rely on autophagy to promote cell survival in the context of ER stress

**Fig 7:**
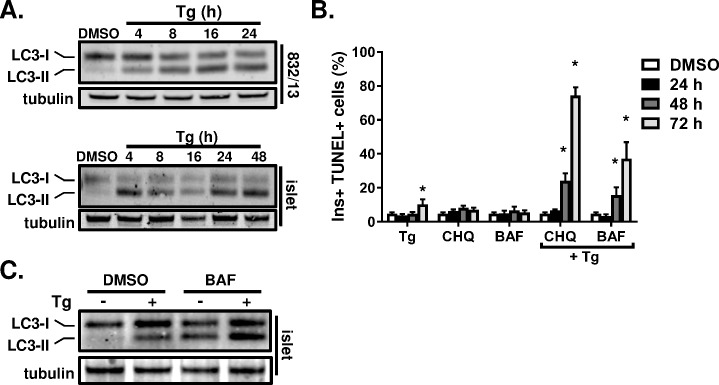
Autophagy inhibition unveils apoptotic response to ER stress. 832/13 (**A**) and primary rat islets (**B**) were treated with DMSO (control) or thapsigargin (100 nM or 1 μM, respectively) for the indicated times. Clarified lysates were examined by immunoblot analysis. LC3-I indicates the full length protein and LC3-II indicates the cleaved form of the protein conjugated to phosphatidylethanolamine. (**B**) Primary rat islets were dispersed onto HTB9 coated 96-well plates and treated with DMSO (control; 96 h), chloroquine (CHQ; 5 μM), or bafilomycin A1 (BAF; 100 nM), alone or in combination with thapsigargin (Tg; 1 μM) for the indicated times. Islet cells were stained for TUNEL and insulin and counterstained with DAPI. Cells were imaged using a high content imager and analyzed using Cellomics software. Data represent the mean +S.E.M. of 3 independent experiments. * p < 0.05 as compared to the treatment with thapsigargin alone for the same time period. (**C**) Rat islets were treated with either DMSO (control) or thapsigargin (Tg; 1 μM) for 24 h and bafilomycin A1 (BAF; 10 nM) was added during the final 6 h. Clarified lysates were examined by immunoblot analysis.

We next considered the possibility that activation of a survival pathway following cell stress may impede progression toward apoptosis and allow functional recovery. Recent evidence indicates that autophagy is an important survival mechanism in the β-cell that counteracts physiological demands associated with insulin resistance and T2D onset [19–21]. Here, we examined 832/13 cells and primary rat islets for activation of autophagy in response to ER stress using conversion of LC3-I into LC3-II via cleavage and subsequent conjugation to phosphatidylethanolamine as an established marker. As shown in Fig 7A, we observed rapid (within 4 h) conversion of LC3-I to LC3-II upon thapsigargin treatment in both 832/13 cells and primary rat islets, consistent with previous reports linking ER stress and chronic palmitate exposure to autophagy activation [34–36]. Furthermore, the sustained accumulation of LC3-II for up to 48 h in primary rat islets suggested that an autophagic response was maintained during prolonged ER stress. Importantly, treatment of islet cells with the autophagy inhibitor bafilomycin A1 either alone or in concert with thapsigargin resulted in further increases in LC3-II levels confirming an active process of autophagy (Fig 7C). Despite evidence of thapsigargin-induced LC3-I to LC3-II conversion in 832/13 cells (Fig 7A), we did not pursue autophagy as a survival mechanism in these cells due to the rapid increase in cell death in response to ER stress (Figs 1 and 2).

We next investigated whether inhibition of autophagy may impact β-cell survival either directly or following chronic ER stress using two distinct lysosomal inhibitors, chloroquine and bafilomycin A1, which prevent formation of autophagosomes. Consistent with our previous data, thapsigargin treatment resulted in a modest increase in the number of TUNEL-positive β-cells following a 72 h treatment (Fig 7B). Similarly, chloroquine and bafilomycin A1 treatment alone modestly induced β-cell apoptosis. In contrast, the inhibition of autophagy in the context of ER stress resulted in a profound increase in TUNEL-positive β-cells within 48 h. By 72 h, treatment with either chloroquine or bafilomycin A1 in combination with thapsigargin increased the number of TUNEL-positive β-cells to greater than 40% and 70%, respectively, approaching the levels observed in insulinoma cells with thapsigargin alone (Fig 2C). Furthermore, treatment of islets with chloroquine during recovery from a 24 h exposure to ER stress also resulted in increased islet cell death (data not shown). These data suggest that primary β-cells rely on an autophagic survival mechanism to prevent ER stress-induced apoptosis.

## Discussions

Type 2 diabetes (T2D) is defined by hyperglycemia and caused by peripheral insulin resistance and insulin insufficiency. Loss of islet β-cell mass and β-cell dysfunction are key events in the progression of insulin resistance towards overt diabetes [1]. A major goal in the development of current diabetes therapies is the identification of islet β-cell pathways that could be exploited to correct and/or enhance islet cell function. Tantamount to this approach is the preservation of islet β-cell mass via activation of islet cell survival pathways.

In this report, we investigated the progression of β-cell death in primary rat islets and insulinoma cells and uncovered a previously unrecognized islet cell survival mechanism that is absent in insulinoma cell lines. In primary islets and insulinoma cells, ER stress activates a strong stress response program as indicated by the upregulation of XBP-1(s), BiP, CHOP, GADD34 and other factors. However, unlike insulinoma cells, upregulation of the stress response program in islets was disconnected from the terminal apoptotic cascade of caspase activation resulting in a considerable delay in the onset of apoptosis, which likely has important physiological consequences. Here we propose that adult islet cells have disengaged the apoptosis program as a protective measure to prevent unnecessary loss of islet cell mass. Given the low replication index of adult β-cells [6, 7], maintenance of a critical cell mass via cell replacement is unlikely to maintain adequate β-cell numbers following cellular insult. Thus, dampening the apoptotic response may be an important survival mechanism in the context of human T2D progression, where β-cell mass declines over the course of decades. Consistent with data presented here, a recent report concluded that primary islet cells do not engage in apoptotic pathways that directly mirror observations in cell culture models [33]. Moreover, the duration of apoptosis defined as the time between initiation (first detectable event) and loss of membrane integrity (final step) was greatly extended (up to 2 days) in β-cell populations.

Reports in post-mitotic cells, such as sympathetic neurons and cardiomyocytes, demonstrate a prolonged delay in apoptosis induction, which is linked to low expression of Apaf-1 [9–11]. In addition, the stoichiometry of XIAP and caspase 3 expression is proposed to be an additional aspect of the neuronal survival mechanism [10, 37]. While we also observed reduced expression of Apaf-1 in primary islets, our data using Apaf-1 overexpression suggest that the level of Apaf-1 does not dictate the apoptotic delay in islets. Moreover, overexpression of caspases 3 and 9 in concert with Apaf-1, which should sufficiently overwhelm IAP expression, was unable to induce apoptosis in primary islets. While these experiments define a remarkable resistance of primary β-cells to the action of classical apoptosis mediators, we still do not understand how islet cells prevent caspase activation. Further examination of other apoptosis checkpoints, such as the maintenance of mitochondrial integrity, may prove valuable in advancing our understanding of this islet survival mechanism.

Our study also uncovered a role for autophagy activation in promoting β-cell survival in the context of ER stress. Here, we show that ER stress activates a sustained autophagic response in primary islet β-cells as previously observed with palmitate treatment [35]. We further show that inhibition of autophagosome formation via bafilomycin A1 or chloroquine in primary islets greatly increased ER stress-induced β-cell death, which has been previously shown in immortalized β-cells [38], but not in adult primary islets as shown here. Consistent with our data, genetic inactivation of autophagy via loss of Atg7 sensitizes primary β-cells to ER stress; however, the full extent of this effect was not realized due to the short duration (24 h) of ER stress exposure [39]. Here, we propose a two-stage model whereby primary β-cells have disengaged key facets of the apoptotic machinery resulting in a significant lag between the (early) induction of stress response and the (delayed) onset of committed apoptosis. During this time lag, an autophagic program may be activated as part of the stress response to mitigate cell damage perhaps through an IRE1/JNK-dependent signaling and/or PERK activation of Atg12 synthesis as observed in other cell systems [34, 36, 40]. In support of this, inhibiting autophagy does not shift the onset of apoptosis to directly follow stress response activation; rather autophagy inhibition only increases the extent of cell death following prolonged stress. Furthermore, we demonstrate that islets can recover function following an acute (24 h) ER stress-induced injury as previously demonstrated for acute cytokine treatment [41] suggesting that islets cells require prolonged stress to commit to apoptosis. While it remains possible that the apparent recovery of β-cell function from ER stress was driven instead by the (early) loss of a small population of dysfunctional islet cells, a sufficient mass of β-cells was retained following ER stress that was able to demonstrate full secretory function. Although 832/13 cells also exhibited signs of autophagy stimulation, the rapid activation of the apoptotic cascade likely precludes any salutary effects of the autophagy program.

As stated, our model suggests that delayed apoptosis and autophagy are two independent mechanisms coordinating islet cell survival; however, our data do not yet rule out the possibility that autophagy may also play a more direct role in preventing late stage apoptosis induction such as preventing cytochrome c release or inhibiting caspase 3 activation. Further studies are needed to dissect the mechanisms of ER stress-induced autophagy activation in islets and to define the role(s) of autophagy in remediating cellular damage. Regulation of mitochondrial integrity may be of crucial importance to both preventing apoptosis induction and allowing functional recovery of β-cells. Recent reports have highlighted the importance of mitophagy in retaining β-cell function in the context of cell stress [42–44] that may be prudent to the models described here. Future studies assessing human islet β-cell apoptotic responses will be crucial to highlighting aspects of this survival pathway relevant to human disease. Our data suggest that the resilience of the islet β-cell offers continued opportunities for the development of diabetes therapies that promote β-cell survival.

## Supporting information

S1 Fig Propidium iodide staining demonstrates low levels of apoptosis in primary rat islets. 832/13 cells (**A**) and primary rat islets (**B**) were treated with DMSO (control), thapsigargin (100 nM or 1 μM, respectively) for the indicated times. (**A**, **B**) Cells were stained with propidium iodide and counted by flow cytometry. Data represent the mean +S.E.M. of 3 independent experiments.
(TIF)

## Abbreviations

BAF: Bafilomycin A1
CHOP: C/EBP homologous protein
CHQ: chloroquine
Etop: etoposide
IAP: inhibitor of apoptosis
PARP: poly (ADP-ribose) polymerase
Tg: thapsigargin
TUNEL: terminal deoxynucleotide transferase-mediated 2’-deoxyuridine 5’-triphosphate nick-end labeling
T2D: Type 2 diabetes
