# Supplementary figures and images for: Delayed apoptosis allows islet β-cells to implement an autophagic mechanism to promote cell survival
Source: PLoS One. 2017 Feb 17;12(2):e0172567. doi: 10.1371/journal.pone.0172567 (PMC5315295; doi:10.1371/journal.pone.0172567)

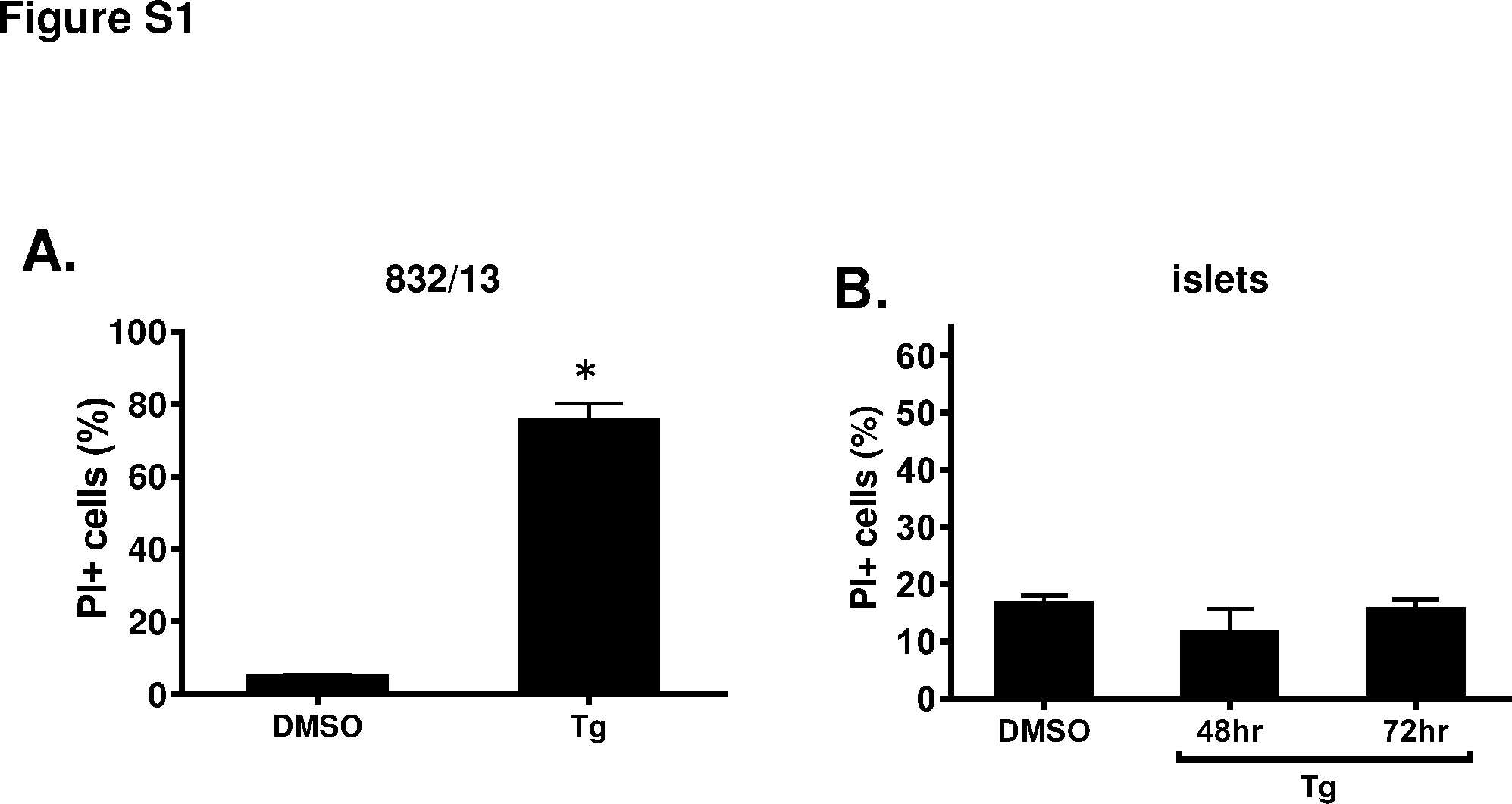

Supplement: S1 Fig — 832/13 cells (A) and primary rat islets (B) were treated with DMSO (control), thapsigargin (100 nM or 1 μM, respectively) for the indicated times. (A, B) Cells were stained with propidium iodide and counted by flow cytometry. Data represent the mean +S.E.M. of 3 independent experiments. (TIF) [file pone.0172567.s001.tif]
